# Commentary: Physical time within human time

**DOI:** 10.3389/fpsyg.2023.1095221

**Published:** 2023-07-31

**Authors:** Marc Wittmann

**Affiliations:** Institute for Frontier Areas of Psychology and Mental Health, Freiburg, Germany

**Keywords:** time perception, passage of time, present moment, neurological, philosophical

Phenomenal consciousness can be viewed as an island of presence (what is happening right now) in the continuous flow of events over time (Metzinger, 2004). This phenomenal characterization encompasses two complementary elements, namely the experience of presence and the sense of dynamic flow. In their dualistic notion of manifest time, Gruber et al. (2022) questioned the verity of these two experiences and concluded that they are illusory, i.e., there is no unique (moving) present, and the dynamic flow of time is rather the existence of a series of discrete snapshots instead of the smooth motions we perceive.

In this study, I will briefly discuss two issues brought up by the authors regarding (1) the illusory nature of the present moment and (2) the illusory nature of the flow. I maintain that we must talk about the veracity of a unique present moment as well as the biological functionality regarding the perception of the dynamic passage of events. Phillips (2014) argued that the temporal structure of experience mirrors the temporal structure of the world. Events in the world unfold in time, and experience mirrors this temporal passage within an extended subjective present (Dorato and Wittmann, 2020).

## The concept of the illusory nature of the present moment

The authors present Hartle's information gathering and utilization system (IGUS) and provide empirical evidence for the theory that an IGUS robot could experience different present times if a split visual screen conveyed both a present event and simultaneously a recent past event. This system would enable the robot to experience the same present twice, which would defy the notion of a unique present. The authors discuss subjects' reports when wearing virtual reality (VR) headsets projecting split screens. Participants in their study claimed to experience a previously presented event (the same event presented twice) “just as real as the first time,” and they felt like they were experiencing “being there,” allegedly an experience of presence.

I want to make a distinction between the subject and the object of perception. The perceived object was first projected to split screen 1 and then to split screen 2 (two events in sequential order). The viewer first perceived object-event x on split screen 1 (as present) and then on split screen 2 (again as present). The object appeared twice on the two screens, but sequentially, within a unique (moving) present. Object-event x appeared to occur in a unique present in both split screens, a unique present at t_1_ and a unique present at t_2_; only the event as object changed its temporal–spatial position, as at t_2_ the subject was aware (knew from short-term memory) that the object in split screen 2 had just been shown in split screen 1 at t_1_. The viewer experienced the same event twice sequentially but in a unique subjective single (sliding) present. When subjects were allowed to switch back and forth between “past” and “present” screens by pressing a button, they perceived the event as subjectively present in the past screen and, when changing to the present screen, also perceived it as in the subjective present, the moving (sliding) present^1^. Importantly, with this notion of the present moment, I do not imply an “objective present moment” as theorized in physics. I am referring to the subjective sense of the present moment, the experience of nowness.

## The concept of the illusory nature of time passage

The authors distinguish between an “illusory change” and a “non-illusory” (completed) change. The first experience relates to events actually happening (dynamic change); the second relates to not having seen the change actually happen. The latter experience was only deduced after a completed change had occurred. While the first experience relates to our everyday, apparent experience, such as a car driving past quickly or a ball being thrown, the second experience is mainly derived from experimental results. Systematic manipulation of dynamically changing or moving stimuli interspersed by different durations of blank inter-stimulus intervals creates subjects' impressions ranging from experiencing dynamic changes (shorter blanks) to not seeing changes actually happening (longer blanks). The former experience represents illusory change; the latter is termed “real” change without the dynamic happening in between. According to the physical notion of a “frozen” block universe or the B-series philosophical model, only the perception of completed change in the order of events is considered real. Perceived dynamic change is merely a perceptual addition, subjectively “painted on” the frames of underlying slow, discrete processing mechanisms underlying the real change in events.

What I contradict is the authors' concept that the apparent dynamic motion of change simply augments experience and has no functional use, that these additions “do not necessarily provide significant information for the observer other than to indicate […] that there are multiple events of unspecified type in between two temporally adjacent stimuli” (Gruber et al., 2022; p. 6). The question is whether an experience governed by neural processes should have some important biological and psychological function. Why has the perception of apparent happening (as dynamically experienced) developed when “real change” is functionally sufficient? The authors claim that certain patients with brain damage have problems perceiving apparent motion but are otherwise not functionally impaired and still receive all essential information.

Individual neurological cases exist in which perceived movement breaks down and patients are functionally affected. One patient with posterior brain damage after an ischemic cerebral infarct was unable to detect continuous visual movement and change (Zihl et al., 1983). Gruber et al. (2022) maintained that the patient had probably lost her ability to perceive apparent motion but still perceived real motion. For example, she had problems properly pouring coffee into a cup because she could not perceive the steady rise of liquid in the cup. She could consequently not find the right moment to stop pouring the coffee. She also reported problems crossing a street with ongoing traffic: “When I'm looking at the car first, it seems far away. But then, when I want to cross the road, suddenly the car is very near” (Zihl et al., 1983; p. 315). This description approximates the notion of real time described by Gruber et al. because it seems as if the patient perceived a series of stills at disparate time points without anything happening in between, thus lacking the impression of a smooth flow of events. This patient reported a severe functional impairment. Such extreme cases are rare. A variety of subjective time distortions has been collected in patients with brain damage of different etiologies which confirm that disruptions in the perception of time passage can create massive functional problems (Blom et al., 2021). The perception of the dynamic happening of events seems to be a prerequisite for proper functioning.

## Author contributions

The author confirms being the sole contributor of this work and has approved it for publication.

## Footnotes

^1^A remark on these theoretical deliberations: virtual reality (VR) is a powerful tool to test and alter experience. Anyone who comments theoretically on the article by Gruber et al. (2022) ideally should have had the experience with the VR headset.
